# CottonFabricImageBD: An image dataset characterized by the percentage of cotton in a fabric for computer vision-based garment recycling

**DOI:** 10.1016/j.dib.2024.110712

**Published:** 2024-07-06

**Authors:** Nishat Tasnim Niloy, Md. Rayhan Ahmed, Sinthia Sarkar Ananna, Sanjida Kater, Iffat Jahan Shorna, Sadika Islam Sneha, Md. Hasanul Ferdaus, Mohammad Manzurul Islam, Mohammad Rifat Ahmmad Rashid, Taskeed Jabid, Md. Sawkat Ali

**Affiliations:** a Department of Computer Science and Engineering, East West University, Aftabnagar, Dhaka, Bangladesh; b Department of Computer Science and Engineering, United International University, Bangladesh

**Keywords:** Computer vision, Textile engineering, Artificial intelligence, Image classification

## Abstract

The utilization of computer vision techniques has significantly enhanced the automation processes across various industries, including textile manufacturing, agriculture, and information technology. Specifically, in the domain of textile manufacturing, these techniques have revolutionized the detection of fiber defects and the quantification of cotton content in fabrics. Traditionally, the assessment of cotton percentages was a labor-intensive and time-consuming process that relied heavily on manual testing methods. However, the adoption of computer vision approaches requires a comprehensive dataset of fabric samples, each with a known cotton percentage, to serve as training data for machine learning models. This paper introduces a novel dataset comprising 1300 original images, covering a wide range of cotton percentages across thirteen distinct categories, from 30% to 99%. By employing image augmentation techniques, such as- rotation, horizontal flip, vertical flip, width shift, height shift, shear range, and zooming, this dataset has been expanded to include a total of 27,300 images, thereby enhancing its utility for training and validating computer vision models aimed at accurately determining cotton content in fabrics. Through the extraction of pertinent features from the images of fabrics, this dataset holds the potential to significantly improve the accuracy and efficiency of computer vision-based cotton percentage detection.

Specifications Table

**Table:** 

Subject	Textile Engineering
Specific subject area	Image processing, Computer vision, Pattern Recognition, Artificial Intelligence
Data format	Raw and processed
Type of data	Raw data consisting of images of the size 900p × 1200p having RGB color in JPG format.
Data collection	This dataset comprises a total of 1300 original and 27,300 augmented fabric images, which have been categorized into 13 different percentages of cotton fabric classes. The fabric image classes include 30 %, 40 %, 50 %, 53 %, 58 %, 60 %, 63 %, 65 %, 66 %, 80 %, 95 %, 98 %, and 99 % cotton. Fabric images were captured using a 48 MP (f/2.0, (wide), 1/2.0″, 0.8 µm, PDAF) camera of a smartphone, model Samsung M12. The measurement of fabric data for each percentage was conducted using a thread-counting machine under the supervision of two textile engineering experts. They also helped in data annotation, which has been done manually with the help of the same thread-counting machine.
Data source location	The locations from where the fabric images were collected - 1. 40/1, B.C.C Road, Thatari Bazar. 2. Jamuna Future Park shopping complex. 3. Bashundhara shopping complex. 4. Mouchak Market. 5. Bongo Bazar Market. 6. Islampur Market. City: Dhaka (Latitude: 23.777176, and Longitude: 90.399452)Country: Bangladesh
Data accessibility	Repository name: Mendeley Data [1] Data identification number: 10.17632/3vc56ddjhw.2 Direct URL to data: https://data.mendeley.com/datasets/3vc56ddjhw/2

## 1 Value of the Data

• Traditionally, determining cotton content has been a tedious and unreliable manual process. Existing image datasets have not addressed this well, either focusing on woven fabrics or mixed types entirely. Even datasets specifically for cotton fabrics are limited, being purely textual or offering only a handful of categories. This dataset helps by providing precise cotton content identification, unlike those focused on fabric type or defects. The lack of such a resource has significantly slowed down progress in automating cotton percentage detection. This collection, being one of the first publicly available, fills this critical gap. It empowers researchers to develop AI-powered solutions that can finally manage this challenge effectively. (Y. [2]), (C. F. J. [3]).
• The scarcity of datasets specifically tailored for the analysis of fabric information poses a significant challenge in the realm of image processing, particularly in determining the cotton percentage in fabrics. Such a dataset is crucial for effectively training these models to accurately detect cotton percentages. This dataset, to our knowledge, stands as one of the first publicly available resources dedicated to facilitating cotton percentage detection through the analysis of fabric images [4].
• The successful recycling of garments hinges on a detailed understanding of the cotton fiber content in the fabric. Knowing the precise cotton percentage allows professionals to select the best materials for enhancing the durability of future products. Automation of this determination process via artificial intelligence (AI) offers a promising avenue to reduce manual labor and mitigate human error. By leveraging AI to calculate cotton percentages accurately and efficiently, the textile industry can streamline the recycling process, ensuring that materials are utilized more effectively and sustainably.
• This dataset comprises 1300 original fabric images and 27,300 augmented images, distributed across thirteen classes. As a publicly accessible resource offered at no cost, it stands to significantly empower researchers aiming to advance the field of supply chain management within the textile industry. The availability of such a dataset facilitates the enhancement of artificial intelligence (AI) applications in textile manufacturing by providing a rich basis for research and development.
• This dataset can be useful to other textile manufacturing automation across various stages. From the initial steps of automated cotton picking and consistent yarn spinning in fiber production, to weaving and knitting intricate patterns with high-speed precision on computerized looms and knitting machines, automation ensures quality and efficiency. Automated dyeing and finishing processes achieve consistent color matching, controlled dye application, and precise printing.

## 2 Background

Wool, cotton, and polyester represent the most prevalent fiber materials in textile manufacturing, with cotton standing out due to its versatility, absorbency, and comfort. Sourced from plants, cotton is celebrated for its durability and flexibility, making it a preferred choice for a wide range of textile products [5]. The cotton content significantly influences the overall quality of cotton fabrics, serving as a key metric for assessing their value and utility. In recent years, a growing consumer demand for sustainability and transparency in the textile industry has underscored the importance of clear communication about the materials used in textile products. This shift in consumer priorities has heightened the focus on the quality of cotton fabrics, spotlighting the need for accurate identification and reporting of cotton percentages to meet these evolving expectations.

## 3 Data Description

**Fig. 1:**
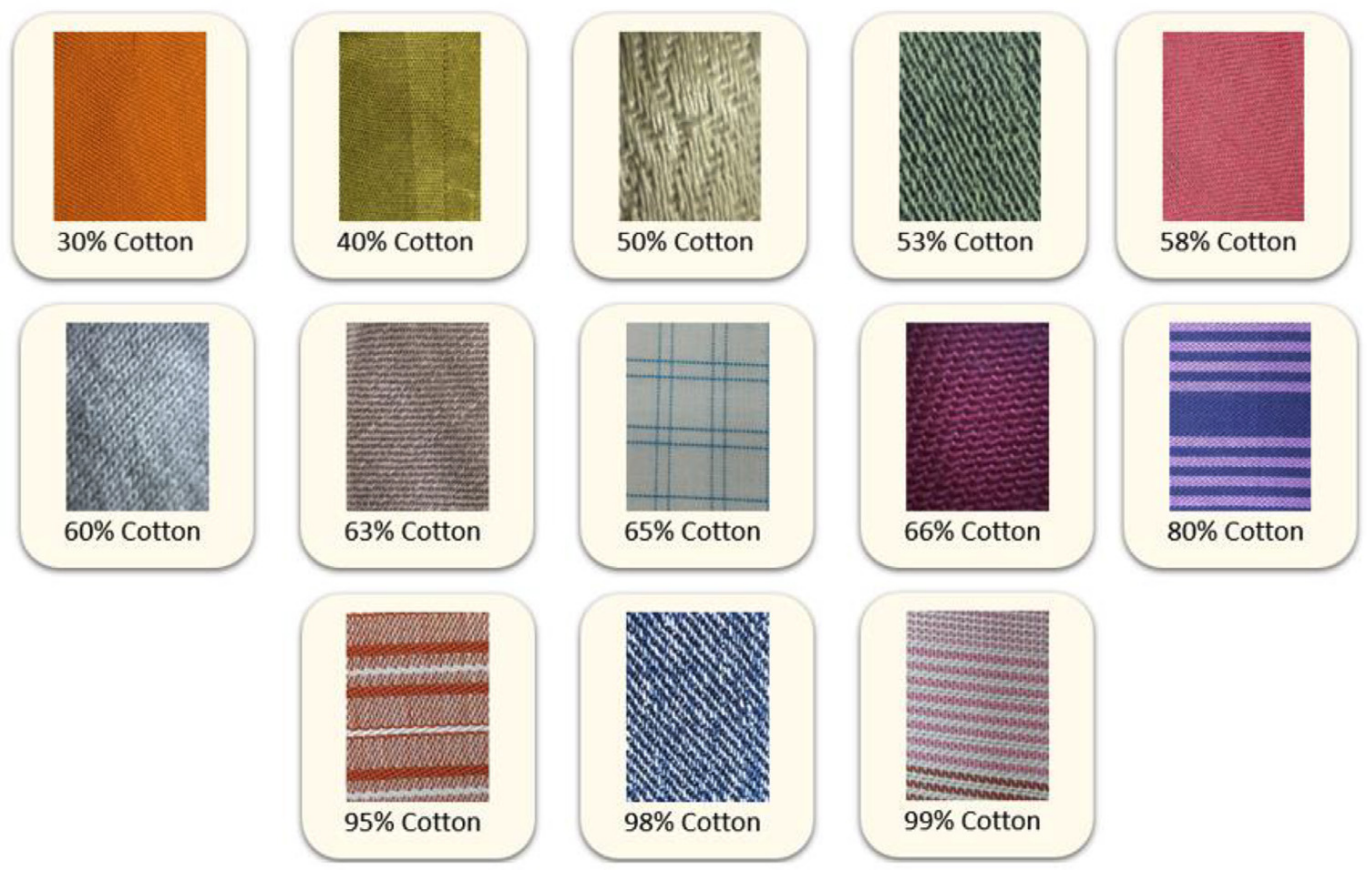
Sample images of the CottonFabricImageBD dataset.

The CottonFabricImageBD dataset contains raw fabric images with different cotton percentages. The cotton percentages are categorized into 13 classes: 30 %, 40 %, 50 %, 53 %, 58 %, 60 %, 63 %, 65 %, 66 %, 80 %, 95 %, 98 %, and 99 %. Fig. 1 shows the fabrics samples from each of the categories. Each of the categories have 100 raw images. To enhance the dataset and provide sufficient instances for training any artificial intelligent models, various image augmentation techniques were applied. These image augmentations contribute to a better convergence of DL-based models [6].

For each of the thirteen categories, 2100 images were generated after augmentation. As a result, the dataset was expanded, resulting in a total of 27,300 images. A summary of the images in the dataset is represented in Table 1. It contains the data type of the image file along with its format, the number of images, the number of classes that are considered to be collected, the number of images in each category, how the data was collected, the locations from where the data was collected and finally where one researcher can use these data.

**Table 1:** Dataset information briefly.

Image Contains	Colored cotton fabric (Dimension: 900p × 1200p)
Format of Files	JPG
Number of Class	13 (30 %, 40 %, 50 %, 53 %, 58 %, 60 %, 63 %, 65 %, 66 %, 80 %, 95 %, 98 %, and 99 %).
Original Image	1300 (In each class: 100)
Augmented Image	27,300 (In each class: 2100)
How data are acquired	The dataset of the percentage of cotton was collected from various sources, including fabric stores, and clothing manufacturers
Where applicable	To automate the percentage of cotton in a fabric measurement especially in garment recycling

**Fig. 2:**
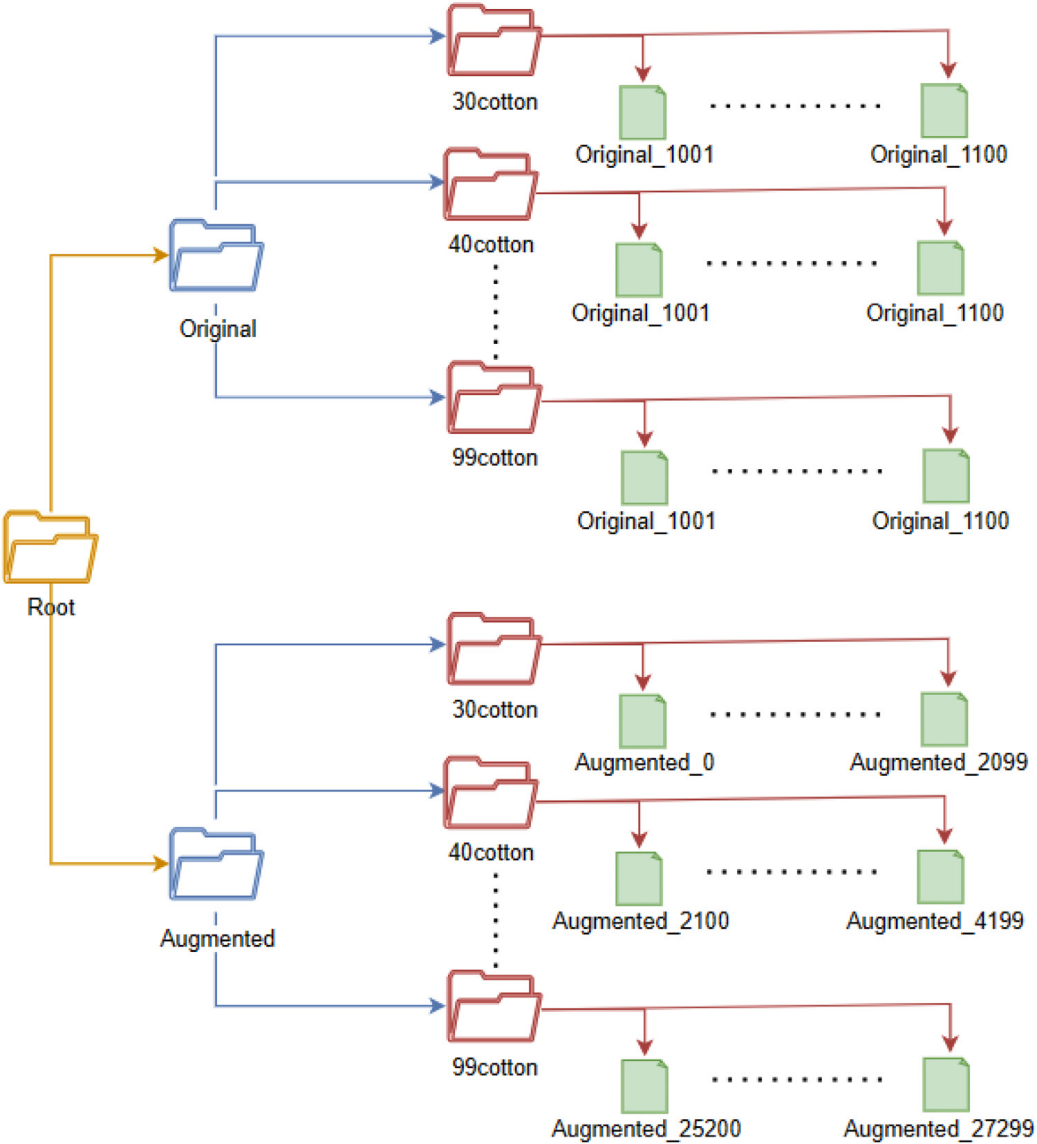
Overview of directories in the Dataset.

The root directory in the repository comprises two main folders, namely “original” and “augmented.” Each of these folders, in turn, contains thirteen sub-folders with the following names: 30cotton, 40cotton, 50cotton, 53cotton, 58cotton, 60cotton, 63cotton, 65cotton, 66cotton, 80cotton, 95cotton, 98cotton, and 99cotton. These sub-folders contain the captured images in JPG format. For the “Original” directory, each of these sub-folders contains 100 images, while in the case of the “Augmented” directory, they contain 2100 images. Fig. 2 illustrates the folder and sub-folder structure that categorizes the images. It is worth noting that this structure is consistent across all folders with similar names, and as such, only a portion of it is depicted in this figure.

## 4 Experimental Design, Materials and Methods

A specialized team, under the guidance of textile manufacturing experts, undertook the meticulous data curation process, emphasizing the importance of initial research to discern cotton patterns in fabrics with a thread-counting machine prior to the actual data collection. A critical phase of the project entailed identifying appropriate fabric stores and manufacturers and obtaining their consent for participation in the research. With these preparatory measures in place, the team proceeded with the systematic collection and processing of data. The comprehensive process for preparing the dataset encompassed several key activities:

• *Data Collection:* The fabric sample images are captured from the chosen locations to create the initial dataset.
• *Measuring the Thread Count:* The thread count was done on the selected fabric samples to assess the quality and density of the fabrics.
• *Cotton Percentage Calculation:* The cotton content was calculated for each fabric sample to classify them according to their cotton percentages.
• *Data Pre-processing:* The collected data was pre-processed for augmentation by standardizing image sizes, enhancing quality, and correcting any discrepancies.
• *Image Augmentation:* Employing image augmentation techniques to expand the dataset, thereby enhancing its diversity and volume for more robust model training.

**Fig. 3:**
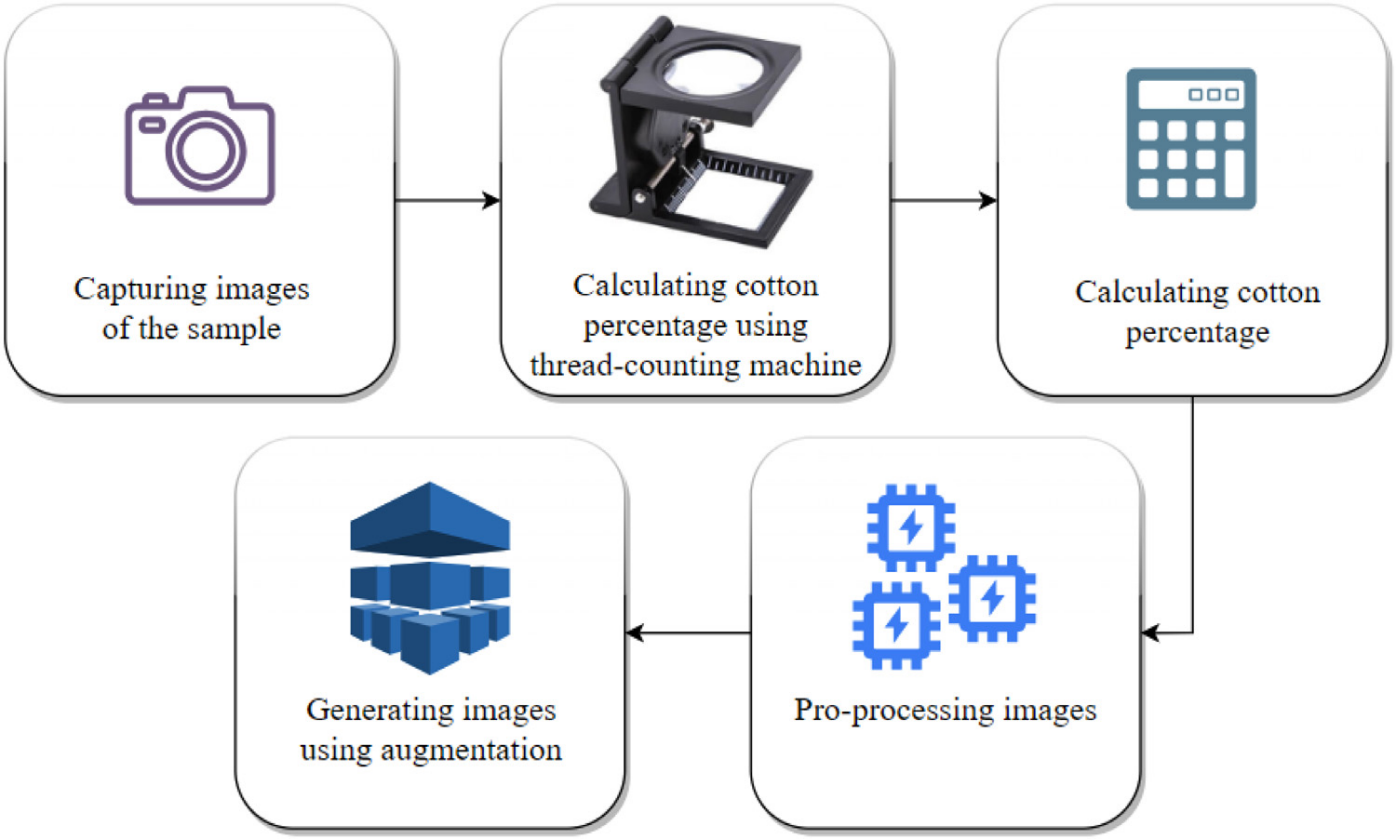
The methodology of dataset preparation.

Fig. 3 shows a flowchart of the methodology of dataset preparation. The following sections describe each of the steps taken to gather the images of this dataset in detail.

### 4.1 Data collection

The default camera settings of the smartphone were used, with automatic exposure lighting. The model of the smartphone that was used to capture the images is Samsung M12, with a 48 MP camera, f/2.0 aperture, (wide), 1/2.0″ sensor size, 0.8 µm pixel size, and PDAF. Fabric samples with cotton content ranging from 30 % to 99 % were selected.

### 4.2 Measuring the thread count

**Fig. 4:**
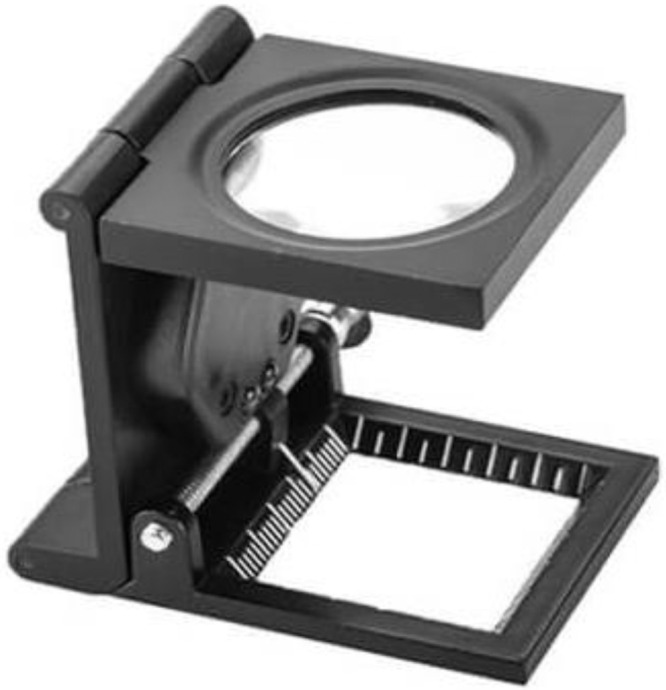
Thread-counting machine.

Images of fabric samples were collected by examining local clothing tags or labels to determine the cotton percentage. After the fabric samples and their labels were collected, a thread counting machine (as shown in Fig. 4) was used to accurately calculate the percentage of cotton, with the assistance of textile industry experts. In the beginning, the thread-counting machine was kept on a plain surface and the threads were aligned in a straight line. A magnifying glass was then placed on the threads and for clear visibility, it was adjusted accordingly. The machine had a counting grid like a ruler with marked lines that helped to track the number of threads.

**Fig. 5:**
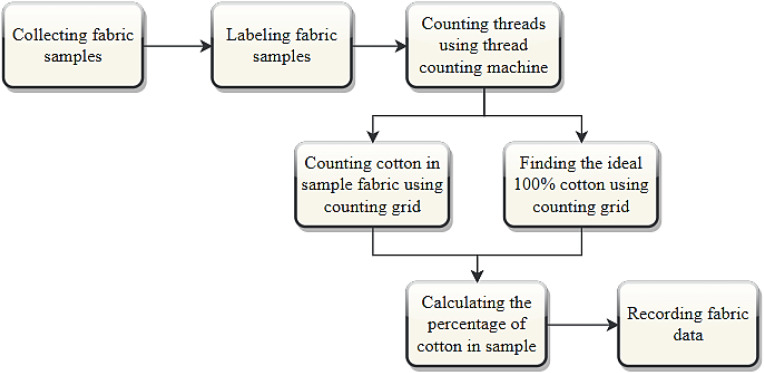
A flowchart to calculate the cotton percentage using a thread-counting machine.

The threads were carefully counted by following them with eyes and using the counting grid to keep a record. Initially, the threads in an ideal fabric sample that consisted of 100 % cotton were counted. The approximate counts for the horizontal and vertical threads were 117 and 73, respectively. Subsequently, the threads in the other fabric samples were counted, and the thread counts were recorded manually. Finally, the percentage of cotton in each fabric sample was calculated. Through this process, data was collected for 13 different percentages of cotton fabric. The total process to calculate the cotton percentage is shown in a flow chart in Fig. 5*.*

### 4.3 Cotton percentage calculation

To categorize the sample into different categories, the percentage of cotton in fabrics must be calculated carefully. The traditional thread count formula that is widely used in the textile industry is used in this research. Following Algorithm 1, the formula to find the cotton percentage can be implemented.

**Table:** **Algorithm 1:** Calculating the Cotton Percentage.

**Input:** horizontal thread count HTC, vertical thread count VTC, horizontal threadcount of ideal fabric iHTC, vertical thread count of ideal fabric iVTC**Output:** cotton percentage CP**Function:** 1: threadCount ← HTC × VTC 2: idealThreadCount ← iHTC × iVTC 3: cottonRatio ← threadCount ÷ idealThreadCount 4: CP ← cottonRatio × 100

To calculate the cotton percentage in a fabric sample, a specific formula that considers the thread count of both the sample and an ideal fabric is employed. For instance, if a random fabric sample has a horizontal thread count (HTC) of 78 and a vertical thread count (VTC) of 64, the total thread count for this sample would be the product of HTC and VTC, which equals (78 × 64) 4992. Comparatively, an ideal fabric sample, with a horizontal thread count (iHTC) of 117 and a vertical thread count (iVTC) of 73, would have a total thread count of (117 × 73) 8541.

The cotton percentage is determined by dividing the thread count of the random fabric sample by that of the ideal fabric sample and then multiplying the quotient by 100 %. Using the given numbers:

Cotton Percentage = (4992/8541) × 100 % = 58.44 %

For practical purposes, this percentage is rounded down to the nearest whole number, resulting in a cotton content classification of 58 %.

### 4.4 Data pre-processing

The training dataset has been expanded using data augmentation methods [7,8] to improve the learning curve of the model. To prepare the images for augmentation, those were labelled, resized, and rescaled. The summary of those processes is provided below:

1. *Data Labelling:* It is the process of naming raw data and adding single or multiple significant educational labels to provide information about it. Each image was labelled and organized into folders so that supervised learning could be introduced based on this dataset [9].
2. *Data Resize:* When an image is scaled, the pixel value changes. Any extraneous pixel information will be eliminated when an image is compressed. Generally, this method resizes any image to 256 pixels. [10].
3. *Data Rescale:* The rescale technique scales down an image to a fixed value where the scaling factor might be a single floating-point number or a collection of numbers, one for each axis. The major difference between this process and data resizing is, that it helps to choose the output image format. [10].

### 4.5 Image augmentation

A total of 1300 images were obtained, and 27,300 images were synthetically generated using various image augmentation techniques, i.e., rotation, horizontal flip, vertical flip, width shift, height shift, shear range, and zooming, resulting in several alternate images, expanding the data set. These transformations help neural networks generalize better by exposing them to different viewpoints of the same object or scene. Additionally, these methods simulate changes in the position of objects within the image, enhancing the modelʼs ability to recognize objects in different locations and training models to recognize objects at different scales and distances. This improves the modelʼs ability to handle variations in object sizes. Several renowned research works have addressed these traditional augmentation processes and have shown their efficiency in augmenting data for computer vision tasks [8]. A summary of those processes is provided below:

1. *Rotation:* It is found that employing a tilt of a picture with an angle of 30 produced good results when applying different rotational angles ranging from 0 to 60. Hence, some random image rotations with a rotation angle of [−30, 30] were chosen, where the blank space is padded with white pixels [11].
2. *Horizontal and Vertical Flip:* Horizontal flip augmentation is the process of horizontally flipping the complete rows and columns of an image pixel. After flipping the complete pixels of an image, a horizontal flip is done on the input image, and the image is returned as a result. Vertical flip augmentation is the process of vertically reversing the complete rows and columns of an image pixel. After flipping the complete pixels of an image, a vertical flip is done on the input image, and the image is returned as a result [11].
3. *Width and Height Shift:* The greatest amount of horizontal shift that is applied to the image during augmentation is specified by this parameter. The greatest horizontal displacement in this instance is 20 % of the imageʼs overall width. The greatest amount of vertical shift that is applied to the image during augmentation is indicated by the parameter height shift range is 0.2. The greatest vertical displacement in this instance is 20 % of the image's overall height [9].
4. *Zoom Range:* The maximum amount of zoom (magnification) that is applied to the image during augmentation is specified by this parameter. The maximum zoom in this instance is 20 % of the image's size [9].
5. *Fill Mode:* The method utilized to fill in any pixels that may be lost during the augmentation process is specified by this option. In this dataset, any gaps are filled with the closest pixel value [9]

## Limitations

• Although all the images are captured using the same device and a similar well-lit environment, some of the original images may be a little bit blurry or noisy. This may lead to any model failing to accurately identify and extract relevant features like texture, thread count, and weave patterns, which are crucial for determining cotton percentage.
• To collect the images from various sources, six places were selected. However, there might be still some bias towards specific fabric types for which the model may become skewed and excel at detecting cotton percentages in those fabrics while struggling with others.
• The preliminary selection of the fabric samples was based on their local clothing tags. If the information on the labels is incorrect, the images will also hold some incorrect data, resulting in inaccurate predictions. Ultimately this situation will misguide the model, leading to unreliable cotton percentage estimations in new fabrics.
• Augmentation techniques might create unrealistic variations in the data that do not reflect real-world scenarios. If the augmentation techniques are not carefully chosen or applied inconsistently across the dataset, they can introduce biases towards specific types of distortions. This bias can then lead to inaccurate performance on fabrics with different weave orientations such as, overfitting, validation issues.

## Ethics Statement

The research was conducted in strict accordance with ethical principles, demonstrating a commitment to the highest standards. Throughout the data collection process, no plants or animals were involved. Besides, all the fabric images were captured in the presence of the respective owners of the shops.

## CRediT authorship contribution statement

**Nishat Tasnim Niloy:** Investigation, Writing – original draft, Writing – review & editing. **Md. Rayhan Ahmed:** Writing – original draft, Data curation. **Sinthia Sarkar Ananna:** Writing – original draft, Methodology, Data curation. **Sanjida Kater:** Writing – original draft, Data curation. **Iffat Jahan Shorna:** Writing – original draft, Investigation, Data curation. **Sadika Islam Sneha:** Visualization, Validation. **Md. Hasanul Ferdaus:** Methodology, Data curation. **Mohammad Manzurul Islam:** Conceptualization, Methodology, Investigation. **Mohammad Rifat Ahmmad Rashid:** Writing – original draft, Writing – review & editing, Visualization, Data curation. **Taskeed Jabid:** Project administration, Investigation. **Md. Sawkat Ali:** Conceptualization, Supervision, Visualization, Project administration.

## Data Availability

CottonFabricImageBD (Original data) (Mendeley Data).
